# Lectin BS‐I inhibits cell migration and invasion *via* AKT/GSK‐3β/β‐catenin pathway in hepatocellular carcinoma

**DOI:** 10.1111/jcmm.13320

**Published:** 2017-09-18

**Authors:** Qiang Jian, Zhao Yang, Jian Shu, Xiawei Liu, Jing Zhang, Zheng Li

**Affiliations:** ^1^ Laboratory for Functional Glycomics College of Life Sciences Northwest University Xi'an Shaanxi Province China; ^2^ Laboratory of Tissue Engineering Faculty of Life Science Northwest University Xi'an Shaanxi Province China

**Keywords:** hepatocellular carcinoma, lectin microarray, GRP78, AKT/GSK‐3β/β‐catenin pathway, Glycosylation

## Abstract

Hepatocellular carcinoma (HCC) is most common malignant cancer worldwide; however, the mortality rate of HCC remains high due to the invasion and metastasis of HCC. Thus, exploring novel treatments to prevent the invasion of HCC is needed for improving clinical outcome of this fatal disease. In this study, we identified lectin from Bandeiraea simplicifolia seeds (BS‐I) binds to metastasis‐associated HCC cell surface glycans by a lectin microarray and inhibits HCC cell migration and invasion through downregulating the matrix metalloproteinase 2 (MMP2), matrix metalloproteinase 9 (MMP9) and urokinase‐type plasminogen activator (uPA) production. These effects of BS‐I were mediated by inhibiting the activation of AKT/GSK‐3β/β‐catenin pathway and depended on specificity of lectin BS‐I binding to GalNAc. GSK3β inhibitors rescued BS‐I‐mediated inhibition of migration and invasion of HCC cell. Further, we identified that lectin BS‐I interacts with sGrp78, affects membrane localization of sGrp78 and attenuates the binding of sGrp78 and p85 to inhibit the activation of AKT/GSK‐3β/β‐catenin pathway. Overexpression of Grp78 or P85 rescues BS‐I‐mediated inhibition of migration and invasion of HCC cell. These findings demonstrated for the first time that BS‐I can act as a novel potential drug to prevent the invasion of HCC.

## Introduction

Hepatocellular carcinoma (HCC), the fifth most common cancer worldwide and the second leading cause of cancer death in men of developing country, causes 745,500 deaths each year 1. New therapeutic strategies such as liver resection, transplantation, radiofrequency ablation and transcatheter arterial chemoembolization have been continuously developed and applied to clinical treatment of HCC; however, the prognosis is still very poor due to the invasion and metastasis of HCC 2. Therefore, exploring novel treatments to prevent the invasion of HCC is urgently needed.

Lectins are highly diverse non‐immune origin proteins, which can selectively recognize and reversibly bind to specific free sugars or glycans present on glycoproteins and glycolipids without altering the structure of carbohydrate 3 and have been widely performed to investigate the alteration of protein glycosylation in cancers. Some astonishing discoveries were reported constantly with the aid of lectins, for example, increased branching of N‐glycans 4, unusual sialylation and fucosylation 5 and truncated O‐glycans 6 were observed in cancers and correlated with cancer development. In addition, the reducing core‐3‐derived glycans were be found in metastatic pancreatic cancer 7 and larger branched tri‐ and tetra‐antennary N‐linked glycans were decreasing in the progression of prostate cancer 8.On the other hand, lectins affect vitality, metastatic and autophagy of cancer cells by modulating representative signalling pathways involved in Bcl‐2 family, caspase family, p53, PI3K/Akt, ERK, BNIP3, Ras‐Raf and ATG families by their pharmacological activities 9. Thus, lectins have potential anti‐cancer role in cancer drug discovery.

The glucose‐regulated protein 78 (GRP78) is a traditionally resident protein of the endoplasmic reticulum (ER) and has multifunction in the resistance to chemotherapy agents, proliferation, invasion and metastasis of many human cancers 10, 11, 12. Recently, it was found that cell surface GRP78 (sGRP78) regulates the invasion and metastasis of many human cancers by forming complexes with specific cell surface proteins and regulates signal transduction 13, 14, 15. sGRP78 not only activated the p21‐activated kinase‐2 (PAK2) signalling pathway and facilitated the invasion and metastasis by binding with α2‐macroglobulin in prostate 16, but also promoted the invasion and metastasis of cancer cells by activating the uPA/uPAR protease system in colorectal cancer 17. Additionally, sGRP78 was found to facilitate the invasion of hepatocellular carcinoma cells by regulating EMT process 18. Further, Liu *et al*. 19 developed a monoclonal antibody against sGRP78 and found that the novel antibody suppressed tumour growth and metastasis by PI3K/AKT signalling. Recent studies suggested that sGRP78 forms complexes with P85 and promotes PI3K signalling. Zhang *et al*. reported that breast and prostate cancer cells resistant to hormonal therapy actively promote GRP78 to the cell surface and forms complex with P85. Overexpression of sGRP78 promoted PIP3 formation and PI3K activation. GRP78 mutated at N‐terminus domain resulted in reducing complex formation with p85 and production of PIP3 20. Lin *et al*. 21 found that CHM‐1 suppresses formation of cell surface‐associated GRP78‐p85α complexes, which inhibited PI3K‐AKT signalling and inducing apoptosis of human nasopharyngeal carcinoma cells. These findings indicated that sGRP78 could be an effective therapeutic target for cancer treatment.

In this study, we screened normal liver cell 7702 and four HCC cell lines (Hep3B, MHCC97L, MHCC97H and HCCLM3), which were different in metastatic capacity by a lectin microarray and found lectin BS‐I specifically binds to metastasis‐associated HCC cell surface glycans. Treating MHCC97L and HCCLM3 cells with 1 or 4 μg/ml BS‐I significantly inhibited cell migration and invasion and down‐regulated the matrix metalloproteinase 2 (MMP2), matrix metalloproteinase 9 (MMP9) and urokinase‐type plasminogen activator (uPA) production. Further, AKT/GSK‐3β/β‐catenin pathway, the upstream of MMP2 and uPA, was found to be involved in the inhibition of cell migration and invasion mediated by BS‐I. Finally, we identified that lectin BS‐I interacts with GRP78, affects membrane localization of sGRP78 and attenuates the binding of sGRP78 and p85 to inhibit the activation of AKT/GSK‐3β/β‐catenin pathway. These findings suggested that BS‐I could prevent the migration and invasion of HCC as a novel potential drug.

## Materials and methods

### Antibodies

Antibodies targeting AKT, phospho‐AKT (P‐AKT Ser473), S6, phospho‐S6 (P‐S6 Ser240/244), GSK3β, phospho‐GSK3β (p‐GSK3β Ser9), β‐catenin, uPA, β‐tubulin, phospho‐β‐catenin (P‐β‐catenin Ser33/37/Thr41), phospho‐B‐Raf (P‐B‐Raf Ser445), phospho‐c‐Raf (P‐c‐Raf Ser338), phospho‐MEK1/2 (P‐MEK1/2 Ser217/221) and P85 were purchased from Cell Signaling Technology (Danvers, MA, USA). β‐actin antibodies were from CMCTAG (Milwaukee, WI, USA), and GRP78 and histone H3 antibodies were from Santa Cruz Biotechnology (Santa Cruz, CA, USA). Antibodies targeting B‐Raf, ERK1/2 and phospho‐FAK (P‐FAK Y397) were purchased from Abcam company (Cambridge, MA, USA). Antibodies targeting MMP2, MMP9, MEK1/2, RAS, c‐Raf, Lamin B, FAK and phospho‐ERK1/2 (P‐ERK1/2 Thr202/Tyr204) were from Wanleibio (Shenyang, China). Active Ras antibody was from NewEast Biosciences (King of Prussia, PA, USA). Horseradish peroxidase (HRP)‐conjugated secondary antibodies were from CWBIO (Peking, China).

### Cell culture

HL7702, Hep3B, MHCC97L and MHCC97H cells were obtained from the Cobioer Bioscience Company (Nanjing, China), and HCCLM3 cells were obtained from China Center for Type Culture Collection (Wuhan, China). HL7702 cells were cultured in 1640 medium (Life Technology, Carlsbad, CA, USA), and Hep3B, MHCC97L, MHCC97H and HCCLM3 cells were cultured in high‐glucose DMEM (Life Technologies Corporation, Carlsbad, CA, USA) and supplemented with 10% FBS (Life Technologies Corporation) in a humidified atmosphere of 5% CO_2_. For GSK3β inhibition, MHCC97L and HCCLM3 cells were firstly incubated with 1 or 4 μg/ml BS‐I (Sigma‐Aldrich, St Louis, MO, USA) for 6 hrs, and then, 0.2 μM CHIR99021 (Selleckchem, Houston, TX, USA) or 4 mM LiCl (Sigma‐Aldrich) was added to the medium.

### Lectin microarray screening for surface glycans on cells

The lectin microarray was produced using 37 lectins (purchased from Vector Laboratories, Sigma‐Aldrich and Life Science Research) with different binding preferences covering N‐ and O‐linked glycans. The lectins were dissolved in the manufacturer's recommended buffer containing 1 mmol/l appropriate monosaccharide to a concentration of 1 mg/ml and spotted on the home‐made epoxysilane‐coated slides according to the protocol 9 with Stealth Micro Spotting Pins (SMP‐10B) (TeleChem, Sunnyvale, CA, USA) by a Capital smart microarrayer (CapitalBio, Beijing, China). Each lectin was spotted in triplicate per block with triplicate blocks on one slide. The slides were incubated in a humidity controlled incubator at 50% humidity overnight and then put in vacuum dryer for 3 hrs at 37°C to allow the lectin immobilization. After incubation, the slides were blocked with the blocking buffer containing 2% BSA in 1× PBS for 45 min. and then rinsed twice with 1× PBST (0.2% Tween 20 in 0.01 M phosphate buffer containing 0.15 M NaCl, pH 7.4), followed by a final rinse in 1× PBS. The slides were dried by centrifugation at 500 g for 5 min. before their use. Then, the cells were harvested by trypsin digestion and washed with PBS for three times. Then, the cells were fixed with 3% glutaraldehyde for 30 min. and washed with PBS for three times. Further, the cells were resuspended in binding buffer (phosphate‐buffered saline (PBS) with 0.5 mM CaCl_2_, 0.1 mM MnCl_2_ and 1% bovine serum albumin (BSA)) and 5 × 10^5^ cells were probed on the lectin microarrays. After incubation for 1 hr at room temperature, the microarrays were washed in PBST (PBS with 0.5% Tween 20). The bound cells were detected by a GenePix 4000B scanner, and the fluorescence intensity of each spot was measured with GenePix Pro 6.0 Software (Molecular Devices, Sunnyvale, CA, USA). Lectins that displayed signal intensities of greater than or equal to three standard deviations above background were defined as positive signals as previously described 22.

### Immunofluorescence microscopy

For lectin staining assay, the cells cultured in 24‐well plates were fixed with 4% PFA for 30 min. on ice. The fixed cells were then incubated with 300 μg/ml Cy3‐ or Cy5‐conjugated ConA, BS‐I and UEA‐I at 4°C overnight. DAPI staining was also carried out on the same sample. For GRP78 and BS‐I staining assay, the fixed cells were firstly incubated with anti‐GRP78 antibody at a 1:100 ratio for 12 hrs, followed by Cy3‐conjugated secondary antibody for 2 hrs. Then, 300 μg/ml Cy5‐conjugated BS‐I was added and incubated at 4°C overnight. Finally, DAPI staining was carried out on the same sample. The results were analysed by microscopy (Nikon, Shinagawa‐Ku, Tokyo, Japan).

### Cell viability assay

The cells were seeded in 96‐well plates at a density of 1 × 10^4^ cells per well. One day later, the medium was changed to serum‐free medium. Triplicate wells were treated with BS‐I at concentrations of 0, 0.5, 1, 2, 4 and 8 μg/ml. The cell viability was assessed using 3‐(4,5‐dimethylthiazol‐ 2‐yl)‐2,5‐diphenyltetrazolium bromide (MTT) following manufacturers’ instructions.

### Transwell assays for cell migration and invasion

The cells were starved in serum‐free medium overnight and harvested by trypsin digestion. Then, the cells were resuspended with serum‐free medium and incubated with BS‐I at concentrations of 0, 0.5, 1, 2, 4 and 8 μg/ml. After that, the cells were added to the upper chamber of transwells with (for invasion assay) or without (for migration assay) matrigel coating in a 24‐well transwell plate (Corning Costar Corp., Cambridge, MA, USA). The cells were allowed to invade or migrate towards medium containing 10% FBS in the lower chambers. After 48‐hrs incubation, all cells that invaded or migrated to the underside of the membrane were fixed and dyed using crystal violet. Ten random fields of view were selected to count the cells, and the mean value was calculated from three independent experiments.

### Transient transfections

Grp78 plasmid was constructed by inserting a full‐length fragment of human Grp78 cDNA with his tag at the N terminal into HindIII and XhoI site of pcDNA3.1 (+) plasmid and P85 plasmid was purchased from Vigene Bioscience (Shandong, China). Transfection was performed according to the instruction of Lipofectamine 2000 (Life Technologies Corporation) transfection protocol. Briefly, MHCC97L and HCCLM3 cells were cultured in high‐glucose DMEM (Life Technologies Corporation) and supplemented with 10% FBS (Life Technologies Corporation). One day prior to the transfection, MHCC97L and HCCLM3 cells were grown in serum‐free medium overnight, and then, 5 μg of Grp78 or P85 plasmids was transfected into MHCC97L and HCCLM3 cells following manufacturers’ instructions, respectively. 24 hrs after transfection, the medium was replaced by serum‐free DMEM medium containing 1 or 4 μg/ml BS‐I (Sigma‐Aldrich).

### Capture of membrane glycoproteins by BS‐I affinity

HCC cells lines MHCC97L and HCCLM3 were cultured in DMEM medium containing 10% FBS, and membrane protein extraction was performed using the Mem‐PER Kit (Pierce, Rockford, IL, USA) with protease cocktail inhibitors (Sigma‐Aldrich) according to standard procedures. Then, 1 mg membrane protein extracted from MHCC97L and HCCLM3 cells was diluted with binding buffer (20 mM Tris‐HCl, pH7.4, 150 mM NaCl, 1 mM MgCl_2_, 1 mM CaCl_2_ and 1 mM MnCl_2_) at a 1:1 ratio. The lysate mixture was then incubated with BS‐I‐coated magnetic particles for 4 hrs at 4°C to capture the membrane glycoproteins, and the captured glycoproteins were released with 8 M urea as described in reference 23.

### Identification of peptides by LC‐Orbitrap MS/MS analysis

The glycoproteins isolated by BS‐I‐coated magnetic particles were concentrated and desalted by a size‐exclusion spin filter (Amicon Ultra‐0.5 3 K device), with a molecular mass cut‐off of 3 kDa. The obtained glycoproteins were denatured in 8 M urea and then deoxidated with 10 mM DTT and carboxyamidomethylated with 20 mM iodoacetamide. Subsequently, trypsin was added at a 1:100 (w/w) ratio of enzyme to protein and the samples were incubated overnight at 37°C. Then, the peptides were desalted using C18 SepPak columns and lyophilized. Finally, the peptides were resolubilized in acetonitrile and analysed using LTQ‐Orbitrap XL ETD mass spectrometers equipped with Easy‐nLC II System (Thermo Fisher, CA, USA) as described in reference 24.

### Western blot analysis

Total protein, membrane protein or nuclear protein was extracted according to the manufacturer's protocol. The protein concentration was quantified using the BCA protein assay. For Western blot, typically 20 μg of whole‐cell lysates was run on 10% Tris‐glycine gradient gel and transferred onto polyvinylidene fluoride membrane (Millipore, Temecula, CA, USA). The membrane was blocked with 5% non‐fat dry milk or BSA in TBS and 0.1% Tween 20 (TBST) for 2 hrs and then incubated with 1 μg/ml primary antibody at 4°C overnight. Membrane was washed three times for 10 min. each and incubated with HRP‐labelled secondary antibody for 2 hrs. After three times wash with TBST, HRP signal was detected using Immobilon Western Chemiluminescent HRP Substrate from Millipore, and the signals were detected by Tanon5200 chemical luminescence imaging system (Tanon, Shanghai, China).

### Immunoprecipitation

For immunoprecipitation, extracted membrane protein was incubated with home‐made magnetic particles conjugated with anti‐GRP78 antibody (Santa Cruz, CA, USA) at 4°C overnight to pull down GRP78 and proteins interacted with it. Magnetic particles were then washed two times with lysis buffer, and the bound proteins were eluted with Laemmli SDS buffer supplemented with 50 mM dithiothreitol, followed by incubation for 10 min. at 100°. Then, the precipitated proteins were analysed by Western blotting.

### Statistics

Tests of significance were conducted using Student's *t*‐test and one‐way ANOVA with Dunnett test, using GraphPad 5.0 software (La Jolla, CA, USA). Values were considered significant at *P* < 0.05.

## Results

### Identification of metastasis‐associated lectin binding to metastatic hepatoma cells

**Figure 1:**
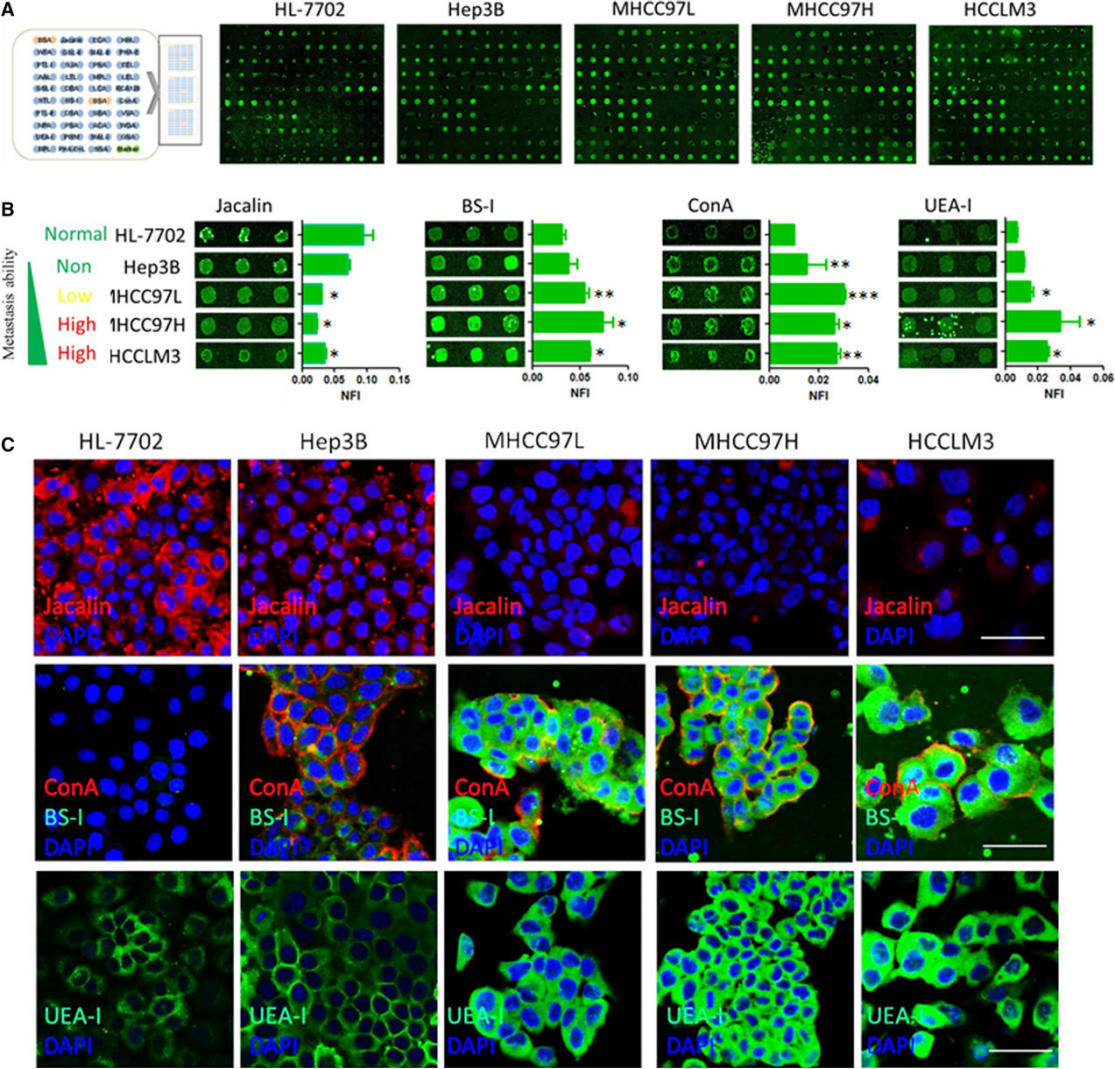
Identification of metastasis‐associated lectin binding to metastatic hepatoma cells. (**A**) Representative lectin microarray binding patterns of the normal liver cell HL7702 and four HCC cells with difference in metastatic capacity. (**B**) Four lectins exhibited different extents of binding to the normal liver cell HL7702 and four HCC cells with difference in metastatic capacity. The indicated intensities are represented as the median values ± standard deviation (S.D.). (**C**) Incubation of Cy3‐ or Cy5‐conjugated Jacalin, ConA, BS‐I and UEA‐I and direct inspection with microscopy further confirmed the cell‐binding tendencies of these lectins, Bar = 50 μm.

Normal liver cell HL7702 and four HCC cell lines (Hep3B, MHCC97L, MHCC97H and HCCLM3), which were different in metastatic capacity, were chosen in this study 25, 26, 27. To identify the cell surface glycan profiles, the harvested cells were firstly fixed by 3% glutaraldehyde and then incubated on 1% BSA blocked lectin microarray containing 37 lectins and each lectin was present in triplicate 28 (Fig. 1A). The relative fold change of each lectin in Hep3B, MHCC97L, MHCC97H and HCCLM3 was summarized in Table 1. As shown in Figure 1B, we found that Lectin Jacalin, BS‐I, ConA and UEA‐I show noticeably different extents of binding to different cell lines. The binding of Jacalin was inversely proportional to their metastatic capacity, while that of BS‐I, ConA and UEA‐I were proportional. Interestingly, with the exception of ConA and UEA‐I that was previously reported to show an enhanced binding to highly metastatic cancer cells 29, 30, BS‐I exhibited stronger binding to the HCC cells of greater metastatic. To verify the results from the lectin microarray, the cells were stained by Cy3‐ or Cy5‐conjugated Jacalin, ConA, BS‐I and UEA‐I. The results of immunofluorescence microscopy were consistent with the lectin microarray. These results indicated that BS‐I indeed bounds strongly metastatic hepatoma cells.

**Table 1:** The relative fold change of each lectin in Hep3B, MHCC97L, MHCC97H and HCCLM3

Lectin	Specificity	Compared with HL7702 (Fold change)			
		Hep3B/HL7702	MHCC97L/HL7702	MHCC97H/HL7702	HCCLM3/HL7702
Jacalin	Galβ1‐3GalNAcα‐Ser/Thr(T), GalNAcα‐Ser/Thr(Tn), GlcNAcβ1‐3‐GalNAcα‐Ser/Thr(Core3), sialyl‐T(ST). not bind to Core2, Core6, and sialyl‐Tn (STn)	0.6555	0.2924*	0.2186*	0.3354*
ECA	Galβ‐1,4GlcNAc (type II), Galβ1‐3GlcNAc (type I)	–	–	–	–
HHL	High‐mannose, Manα1‐3Man, Manα1‐6Man, Man5‐GlcNAc2‐Asn	–	–	–	–
WFA	Terminating in GalNAcα/β1‐3/6Gal	1.5275*	–	–	–
GSL‐II	GlcNAc and agalactosylated tri/tetra‐antennary glycans	0.6084	–	0.653	0.5197
MAL‐II	Siaα2‐3Galβ1‐4Glc(NAc)/Glc, Siaα2‐3Gal, Siaα2‐3, Siaα2‐3GalNAc	–	–	–	0.5271
PHA‐E	Bisecting GlcNAc, biantennary complex‐type N‐glycan with outer Gal	–	–	–	–
PTL‐I	GalNAc, GalNAcα‐1,3Gal, GalNAcα‐1,3Galβ‐1,3/4Glc	–	–	–	–
SJA	Terminal GalNAc and Gal	–	–	–	–
PNA	Galβ1‐3GalNAcα‐Ser/Thr(T)	–	–	–	–
EEL	Galα1‐3(Fucα1‐2)Gal (blood group B antigen)	–	1.8906	2.41	–
AAL	Fucα1‐6 GlcNAc(core fucose), Fucα1‐3(Galβ1‐4)GlcNAc	–	–	–	0.6266
LTL	Fucα1‐3(Galβ1‐4)GlcNAc, anti‐H blood group specificity	–	–	–	–
MPL	Galβ1‐3GalNAc, GalNAc	2.0241	2.0205	–	0.4372
LEL	(GlcNAc)n, high‐mannose type N‐glycans	–	–	–	–
GSL‐I	αGalNAc, αGal, anti‐A and B	1.8765	1.7708	2.4714	2.4426*
DBA	GalNAcα1‐3((Fucα1‐2))Gal (blood group A antigen)	8.1099	5.0606	–	4.587
LCA	α‐D‐Man, Fucα‐1,6GlcNAc, α‐D‐Glc	–	–	–	0.581
RCA120	β‐Gal, Galβ‐1,4GlcNAc (type II), Galβ1‐3GlcNAc (type I)	–	0.6525	0.5005	–
STL	Trimers and tetramers of GlcNAc, core (GlcNAc) of N‐glycan, oligosaccharide containing GlcNAc and MurNAc	–	–	–	–
BS‐I	α‐Gal, α‐GalNAc, Galα‐1,3Gal, Galα‐1,6Glc	–	1.6979**	2.7326*	1.9374*
ConA	Manα1‐6(Manα1‐3)Man, terminal GlcNAc	2.431**	2.9855***	2.5552*	2.7973**
PTL‐II	Gal, blood group H, T antigen	0.5604	–	0.0961	–
DSA	β‐D‐GlcNAc, (GlcNAcβ1‐4)n, Galβ1‐4GlcNAc	–	–	–	–
SBA	(GalNAc)n, GalNAcα1‐3Gal, blood group A	0.0824*	–	0.4721*	–
VVA	GalNAcα‐Ser/Thr(Tn), GalNAcα1‐3Gal	0.0595	1.7224*	–	0.5499
NPA	High‐mannose, Manα1‐6Man	0.4651	–	–	–
PSA	α‐D‐Man, Fucα‐1,6GlcNAc, α‐D‐Glc	–	–	–	–
ACA	Galβ1‐3GalNAcα‐Ser/Thr (T antigen), sialyl‐T(ST) tissue staining patterns are markedly different than those obtained with either PNA or Jacalin	–	–	–	–
WGA	Multivalent Sia and (GlcNAc)n	–	–	–	–
UEA‐I	Fucα1‐2Galβ1‐4Glc(NAc)	1.6503	2.0554*	6.745*	3.7345**
PWM	Branched (LacNAc)n	–	2.6102	1.5043	0.5286
MAL‐I	Galβ‐1,4GlcNAc, Galβ1‐3GlcNAc	0.4427	–	0.5879	0.5813
GNA	High‐mannose, Manα1‐3Man	2.6078*	2.7187*	2.5923	3.8333
BPL	Galβ1‐3GalNAc, Terminal GalNAc	6.5855	3.8312*	9.6256	1.5574
PHA‐E+L	N‐glycans, tri‐ and tetra‐antennary complex‐type N‐glycan	–	–	–	–
SNA	Sia2‐6Gal/GalNAc	–	1.6537**	–	–

Signal intensities obtained for lectin microarrays were normalized, fold changes (≥1.5 and ≤0.67) were calculated; *P < 0.05; **P < 0.01; ***P < 0.0001; –, no significant difference.

### Lectin BS‐I inhibits migration and invasion of HCC cell by suppressing AKT/GSK‐3β/β‐catenin pathway

**Figure 2:**
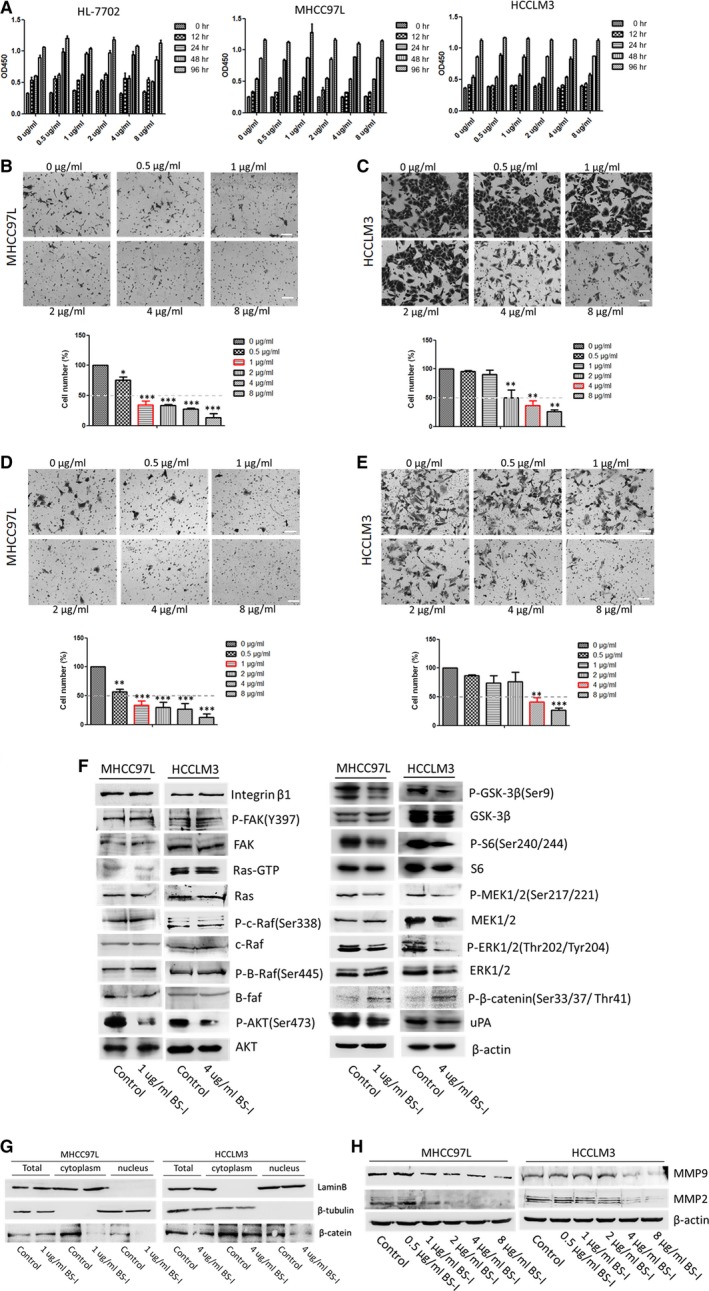
Lectin BS‐I inhibited migration and invasion of HCC cell by suppressing AKT/GSK‐3β/β‐catenin pathway. (**A**) Cell viabilities of normal liver cell HL7702 and four HCC cells with difference in metastatic capacity treated with BS‐I at different concentrations. (**B**) Migration assay for MHCC97L cells treated with BS‐I at different concentrations. Data represent the means ± S.D. from three repeated experiments, *and*** represent *P* < 0.05 and *P* < 0.0001, respectively. (**C**) Migration assay for HCCLM3 cells treated with BS‐I at different concentrations. Data represent the means ± S.D. from three repeated experiments, ** represents *P* < 0.001. (**D**) Invasion assay for MHCC97L cells treated with BS‐I at different concentrations with collagen pre‐coated inserts. Data represent the means ± S.D. from three repeated experiments, **and*** represent *P* < 0.001 and *P* < 0.0001, respectively. (**E**) Invasion assay for HCCLM3 cells treated with BS‐I at different concentrations with collagen pre‐coated inserts. Data represent the means ± S.D. from three repeated experiments, **and*** represent *P* < 0.001 and *P* < 0.0001, respectively. (**F**) Western blot detected the effects of 1 μg/ml and 4 μg/ml BS‐I on the expression of related molecules of RAS/RAF/MEK/ERK, integrin/FAK and AKT/GSK‐3β/β‐catenin pathways in MHCC97L and HCCLM3 cells, respectively. (**G**) Western blot detected the effects of 1 μg/ml and 4 μg/ml BS‐I on β‐catenin expression. (**H**) Western blot detected MMP2 and MMP9 expression in MHCC97L and HCCLM3 cells treated with BS‐I at different concentrations.

To investigate whether lectin BS‐I affects any processes associated with metastasis, normal liver cell HL7702 and two HCC cells with low metastatic potential (MHCC97L) or high metastatic potential (HCCLM3) were chosen and the effects of lectin BS‐I on cell viability were firstly evaluated by MTT assays. As shown in Figure 2A, the viabilities of three cell lines incubated with BS‐I at 0, 0.5, 1, 2, 4, 8 μg/ml from 0 to 96 hrs were not changed. Further, we evaluated the effects of lectin BS‐I on cell migration and invasion by transwell assay. The migration (Fig. 2B and C) and invasion (Fig. 2D and E) capability of MHCC97L and HCCLM3 were inhibited by lectin BS‐I, particularly at the highest lectin BS‐I concentration. And the migration and invasion rate of MHCC97L cells was significantly reduced by 50% at 1 μg/ml BS‐I, while migration and invasion rate of HCCLM3 cells was significantly reduced by 50% at 4 μg/ml BS‐I. This finding suggested that lectin BS‐I recognized specific glycans might play a significant role in the migration and invasion capacity of HCC cells and added BS‐I to block BS‐I recognized specific glycans inhibited the migration and invasion of HCC.

Matrix metalloproteinase 2 (MMP2) and MMP9 have been implicated to play important roles in cancer cell invasion and metastasis 31, 32. Thus, we firstly evaluated whether lectin BS‐I effects the expression of MMP2 and MMP9 by Western blot assay. As shown in Figure 2H, lectin BS‐I reduced the expression of MMP2 and MMP9 in a dose‐dependent manner, and 1 μg/ml BS‐I and 4 μg/ml BS‐I could apparently reduce the expression of MMP2 and MMP9 in MHCC97L and HCCLM3 cells, respectively.

It was reported that RAS/RAF/MEK/ERK, integrin/FAK and AKT/GSK‐3β/β‐catenin pathways regulate the invasion and metastasis of HCC 33, 34. Thus, Western blot assay was performed to detect the expression of related molecules of the three pathways. As shown in Figure 2F, the expressions of integrinβ1, FAK and phosphorylated FAK were not changed after 1 μg/ml BS‐I and 4 μg/ml BS‐I treatment in MHCC97L and HCCLM3 cells. The result indicated that BS‐I inhibits migration and invasion of HCC cell is not *via* integrin/FAK pathways. In addition, BS‐I could not induce significant degradation of active Ras, phosphorylated B‐Raf and phosphorylated C‐Raf in MHCC97L and HCCLM3 cells. However, the protein levels of phosphorylated AKT, phosphorylated GSK3β, phosphorylated S6, phosphorylated MEK1/2 and phosphorylated ERK1/2 were decreased after 1 μg/ml BS‐I and 4 μg/ml BS‐I treatment in MHCC97L and HCCLM3 cells. Moreover, a decrease in β‐catenin nuclear translocation (Fig. 2G) and an increase in phosphorylated β‐catenin were found after BS‐I treatment (Fig. 2F). Finally, uPA, the downstream target of β‐catenin, was decreased after BS‐I treatment. These results indicated that BS‐I inhibits migration and invasion of HCC cell by suppressing AKT/GSK‐3β/β‐catenin pathway.

**Figure 3:**
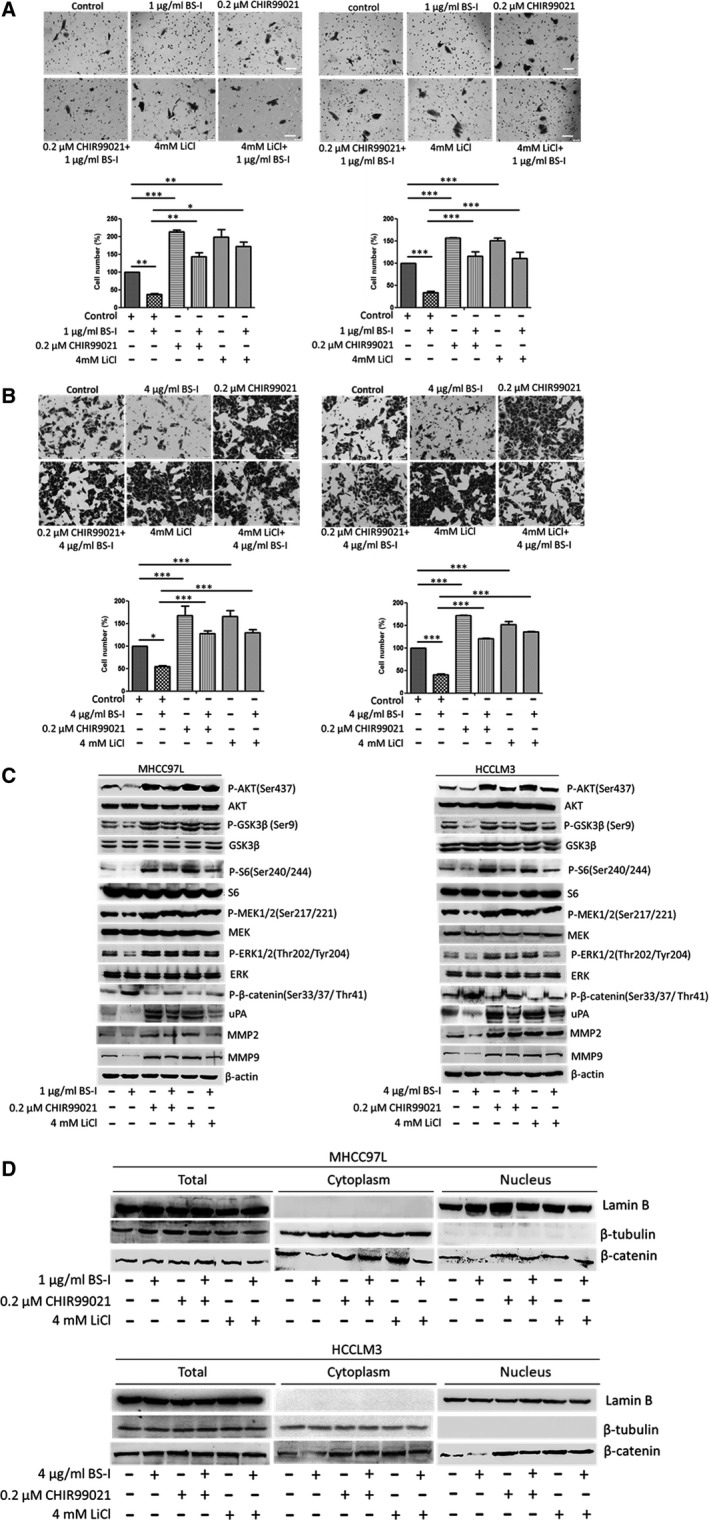
GSK3β inhibitors rescue BS‐I‐mediated inhibition of migration and invasion of HCC cell. (**A**) Migration (left panel) and invasion (right panel) assay for MHCC97L cells incorporated with 0.2 μM CHIR99021or 4 mM LiCl. Data represent the means ± S.D. from three repeated experiments, * represent *P* < 0.05, ** represent *P* < 0.001, *** represent *P* < 0.0001. (**B**) Migration (left panel) and invasion (right panel) assay for HCCLM3 cells incorporated with 0.2 μM CHIR99021or 4 mM LiCl. Data represent the means ± S.D. from three repeated experiments, *and*** represent *P* < 0.05 and *P* < 0.0001, respectively. (**C**) Western blot detected the expression of related molecules of AKT/GSK‐3β/β‐catenin pathways incorporated with 0.2 μM CHIR99021or 4 mM LiCl in MHCC97L and HCCLM3 cells, respectively. (**D**) Western blot detected the β‐catenin expression incorporated with 0.2 μM CHIR99021or 4 mM LiCl in MHCC97L and HCCLM3 cells, respectively.

To confirm our finding, CHIR99021 and LiCl were used to inhibit the activity of GSK3β and protect β‐catenin from degradation. As shown in Figure 3A and B, 0.2 μM CHIR99021 or 4 mM LiCl promoted cell migration and invasion, compared to the control transfected or BS‐I treated group. Importantly, we found that the combination of BS‐I with the GSK3 inhibitor CHIR99021 (0.2 μM) or LiCl (4 mM) resulted in promotion of the migration and invasion of MHCC97L and HCCLM3 cells, compared with BS‐I treatment group. In addition, the results of Western blot assay shown that the expression of phosphorylated AKT, phosphorylated GSK3β, phosphorylated S6, phosphorylated MEK1/2 and phosphorylated ERK1/2 were increased in MHCC97L and HCCLM3 cells, compared with BS‐I treatment group (Fig. 3C). An increase in β‐catenin nuclear translocation (Fig. 3D) and a decrease in phosphorylated β‐catenin (Fig. 3C) were found as well after combination of BS‐I with the GSK3 inhibitor. Further, we found that combination of BS‐I with the GSK3 inhibitor result in an increase in protein levels of uPA, MMP2 and MMP9, compared with BS‐I treatment group. These results indicated that BS‐I inhibits migration and invasion of HCC cell by suppressing AKT/GSK‐3β/β‐catenin pathway.

### Inhibition of migration and invasion of HCC cell mediated by lectin BS‐I requires binding of BS‐I to GalNAc

**Figure 4:**
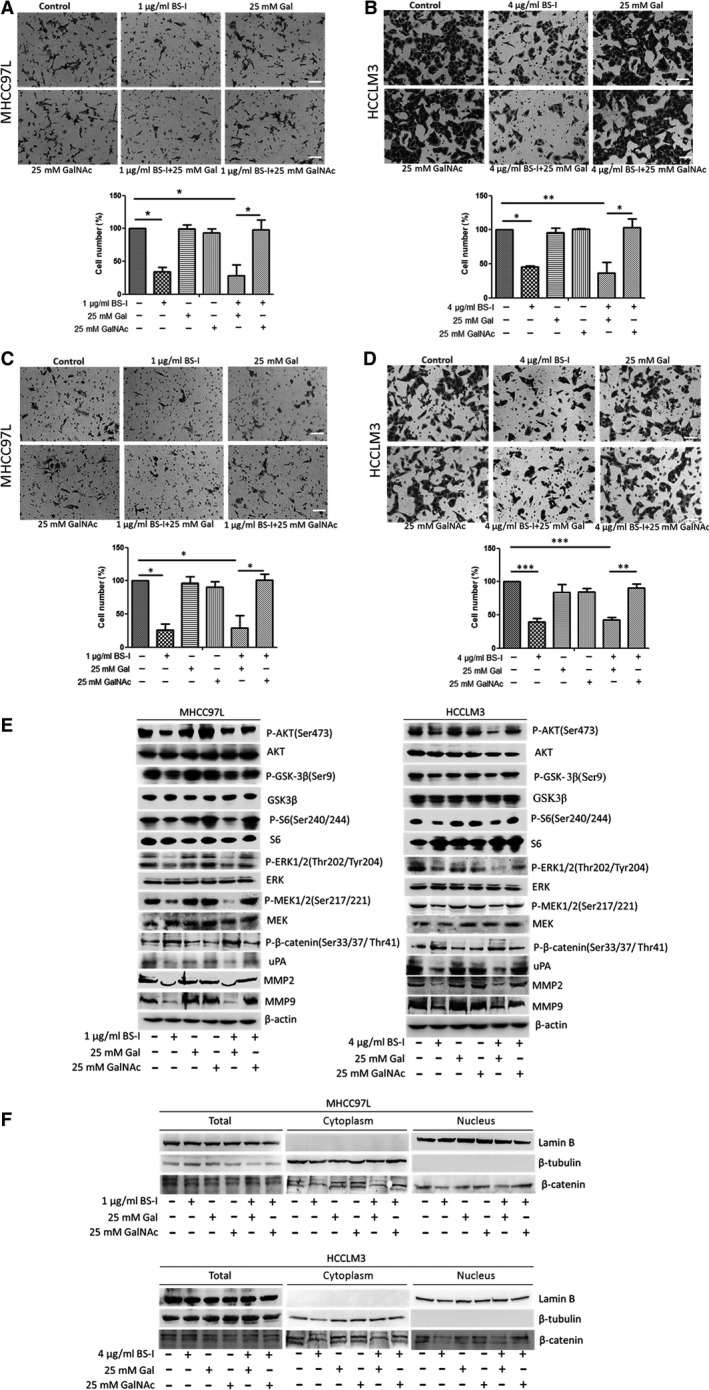
Inhibition of migration and invasion of HCC cell mediated by lectin BS‐I requires binding of BS‐I to GalNAc. (**A**) Migration assay for MHCC97L cells incorporated with 25 mM Gal or GalNAc. Data represent the means ± S.D. from three repeated experiments, * represent *P* < 0.05. (**B**) Migration assay for HCCLM3 cells incorporated with 25 mM Gal or GalNAc. Data represent the means ± S.D. from three repeated experiments, *and** represent *P* < 0.05 and *P* < 0.001, respectively. (**C**) Invasion assay for MHCC97L cells incorporated with 25 mM Gal or GalNAc with collagen pre‐coated inserts. Data represent the means ± S.D. from three repeated experiments, and * represent *P* < 0.005. (**D**) Invasion assay for HCCLM3 cells incorporated with 25 mM Gal or GalNAc with collagen pre‐coated inserts. Data represent the means ± S.D. from three repeated experiments, **and*** represent *P* < 0.001 and *P* < 0.0001, respectively. (**E**) Western blot detected the expression of related molecules of AKT/GSK‐3β/β‐catenin pathways incorporated with 25 mM Gal or GalNA in MHCC97L and HCCLM3 cells, respectively. (**F**) Western blot detected the β‐catenin expression incorporated with 25 mM Gal or GalNA in MHCC97L and HCCLM3 cells, respectively.

Lectin BS‐I is a heterogeneous tetramer of two subunits, one binds only terminal αGal and the other binds αGal and N‐acetylgalactosamine (GalNAc) 35. As shown in Figure 4A–D, inhibition of cell migration and invasion to MHCC97L and HCCLM3 cells by BS‐I was blocked by incorporation of 25 mM GalNAc but not with 25 mM galactose. The results indicated that the inhibition required binding of BS‐I to GalNAc contained on the receptors in MHCC97L and HCCLM3 cells. We then detected the expression of β‐catenin, its downstream target and upstream regulators by Western blot analysis. We found that incorporation of 25 mM GalNAc specifically blocked the inhibition of MMP2 and MMP9 expression by BS‐I (Fig. 4E). Compared with BS‐I treat group, the expression of uPA and β‐catenin (Fig. 4F) in nuclear was significantly increased when 25 mM GalNAc was incorporated with 1 and 4 μg/ml BS‐I in MHCC97L and HCCLM3 cells, respectively. Furthermore, the phosphorylated AKT, phosphorylated S6 and phosphorylated GSK3β levels were increasing, compared to BS‐I treat group (Fig. 4E). Furthermore, the phosphorylated AKT, phosphorylated S6, phosphorylated GSK3β, phosphorylated MEK and phosphorylated ERK levels were increased, compared to BS‐I treat group (Fig. 4E). These results indicated that GalNAc blocked binding of BS‐I to GalNAc contained on the receptors in MHCC97L and HCCLM3 cells and rescued the inhibition of AKT/GSK‐3β/β‐catenin pathway mediated by lectin BS‐I.

### Identification of GRP78 as a BS‐I‐recognized membrane glycoprotein

**Figure 5:**
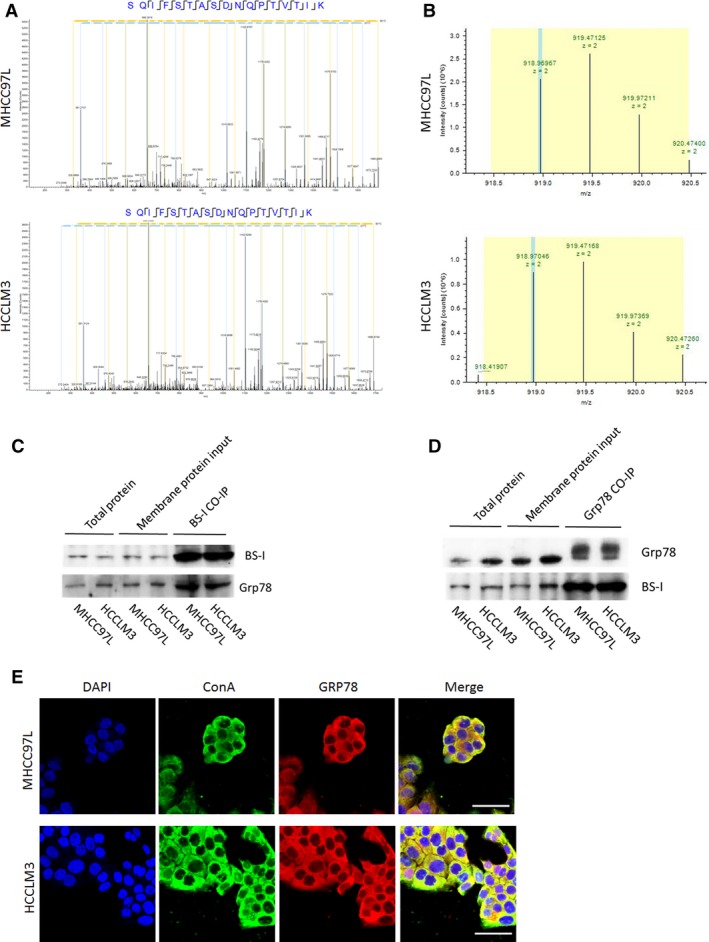
Identification of GRP78 as a BS‐I‐recognized membrane glycoprotein. (**A**) LC‐MS/MS spectrum of a peptide. The sequence of the peptide was identified as SQIFSTASDNQPTVTIK, which was from GRP78. (**B**) Representative base peak chromatogram of GRP78 in the MHCC97L cells (top) and HCCLM3 cells (down). (**C**) BS‐I immuno‐ precipitation assay for MHCC97L and HCCLM3 cells. (**D**) GRP78 immunoprecipitation assay for MHCC97L and HCCLM3 cells. (**E**) Intracellular localization of GRP78 and BS‐I with microscopy, bar = 50 μm.

To identify potential lectin BS‐I recognized specific membrane glycoproteins in the HCC cells, membrane proteins of MHCC97L and HCCLM3 were enriched by BS‐I‐coated magnetic particles, enriched membrane proteins of MHCC97L, and HCCLM3 were performed by LC‐Orbitrap MS/MS analysis. As a result, GRP78 was identified both in MHCC97L and HCCLM3 HCC cells (Fig. 5A and B), which can exist on the plasma membrane and form complexes with specific protein partners, regulating invasion and metastasis of many human cancers 13, 14, 15. In addition, the result of BS‐I immunoprecipitation demonstrated that the protein found in Figure 5A and B was GRP78 (Fig. 5C). To verify the finding above, GRP78 immunoprecipitation and immune‐ fluorescence staining were performed. As a result, GRP78 was found efficiently coprecipitated with BS‐I (Fig. 5D) and co‐localization of GRP78 and BS‐I in cell membrane was found in Figure 5E.

### Lectin BS‐I attenuates the binding of sGRP78 and p85

**Figure 6:**
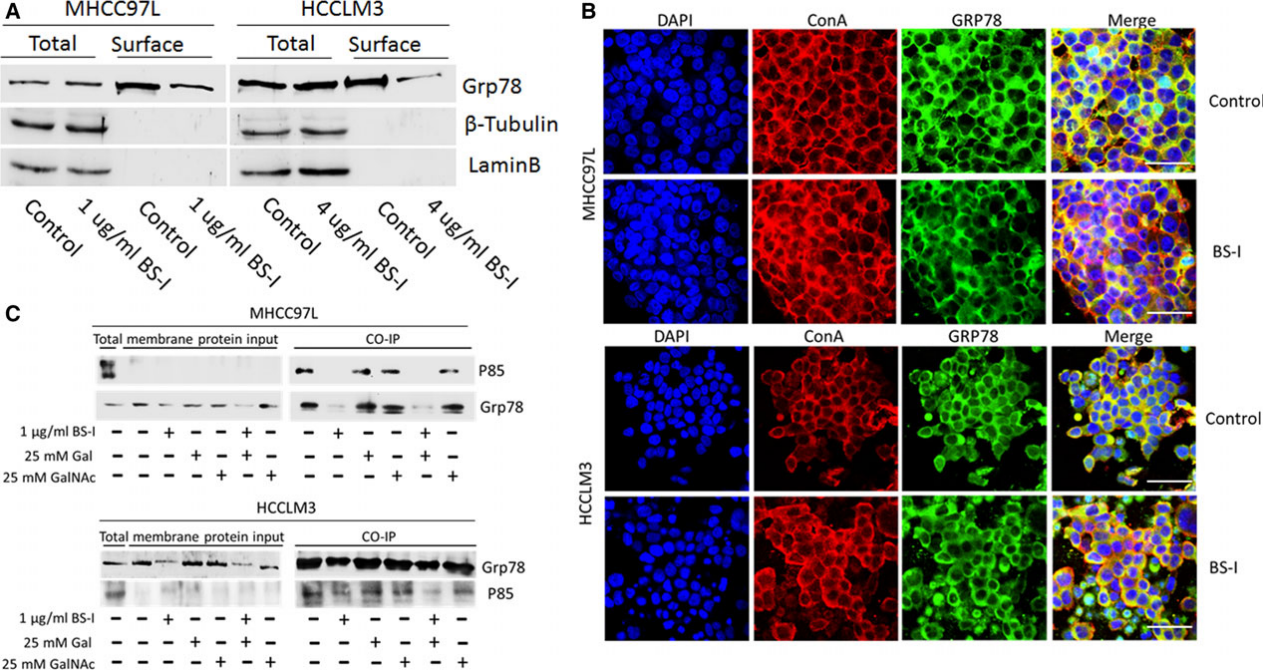
Lectin BS‐I attenuated the binding of sGRP78 and p85. (**A**)Western blot detected the expression of GRP78 in surface and whole cells. (**B**) The alteration of intracellular localization of GRP78 treated by BS‐I with microscopy, bar = 50 μm. (**C**) Co‐immunoprecipitation of GRP78 and P85, using membrane protein from MHCC97L cells treated with 1 μg/ml BS‐I or incorporation with 25 mM Gal or GalNAc.

To illuminate the mechanism that lectin BS‐I inhibited HCC cell migration and invasion and suppressed AKT/GSK‐3β/β‐catenin pathway, we detected the expression of total GRP78 (tGRP78) and surface GRP78 (sGRP78) by Western blot analysis. As shown in Figure 6A, treatment of MHCC97L or HCCLM3 cells using 1 and 4 μg/ml BS‐I reduced the expression of sGRP78, respectively. The similar results were found by immunofluorescence staining (Fig. 6B). Recent studies suggested that sGRP78 forms complexes with P85 and promotes PI3K signalling 20, 21. Thus, we further evaluated whether BS‐I affects the binding of sGRP78 and p85 by immunoprecipitation assay. As shown in Figure 6C, the binding of sGRP78 and p85 was attenuated by 1 μg/ml BS‐I and 4 μg/ml BS‐I in MHCC97L or HCCLM3 cells, respectively. The results indicated that BS‐I inhibited HCC cell migration and invasion and suppressed AKT/GSK‐3β/β‐catenin pathway by attenuating the binding of sGRP78 and p85.

**Figure 7:**
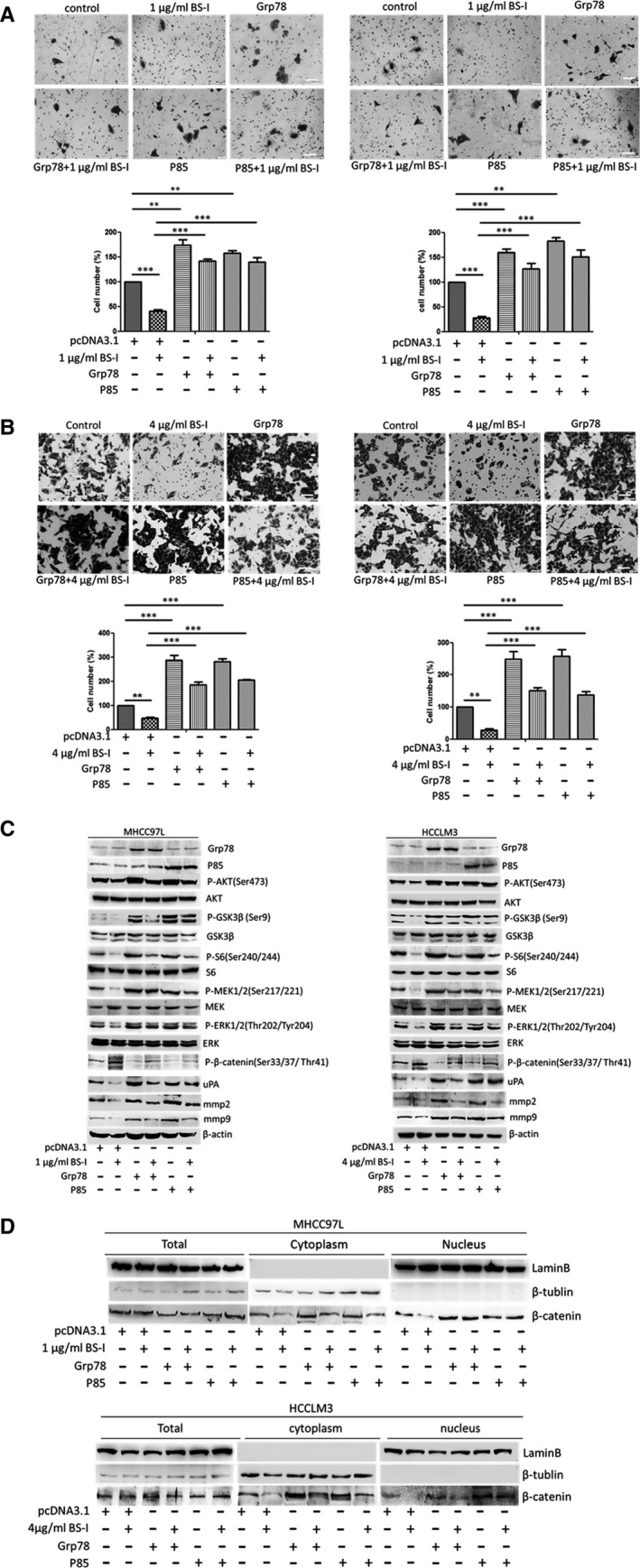
Overexpression of Grp78 or P85 rescues BS‐I‐mediated inhibition of migration and invasion of HCC cell. (**A**) The effect of combination of over‐expression of Grp78 or P85 with BS‐I on migration (left panel) and invasion (right panel) of MHCC97L cells. Data represent the means ± S.D. from three repeated experiments, ** and *** represent *P* < 0.001 and *P* < 0.0001, respectively. (**B**) The effect of combination of overexpression of Grp78 or P85 with BS‐I on migration (left panel) and invasion (right panel) of HCCLM3 cells. Data represent the means ± S.D. from three repeated experiments, ** and *** represent *P* < 0.001 and *P* < 0.0001, respectively. (**C**) Western blot detected the effect of combination of overexpression of Grp78 or P85 with BS‐I on the expression of related molecules of AKT/GSK‐3β/β‐catenin pathways in MHCC97L and HCCLM3 cells, respectively. (**D**) Western blot detected the effect of combination of overexpression of Grp78 or P85 with BS‐I on the β‐catenin expression in MHCC97L and HCCLM3 cells, respectively.

We further transiently transfected MHCC97L and HCCLM3 cells with Grp78 or P85 plasmids to investigate whether overexpressions of Grp78 and P85 rescue the BS‐1‐suppressed‐AKT/GSK‐3β/β‐catenin pathway. The results showed that MHCC97L and HCCLM3 cells transiently transfected with Grp78 or P85 plasmid exhibited promoted cell migration and invasion, compared to the control transfected or BS‐I treated group (Fig. 7A and B). Interestingly, we found that the combination of overexpression of Grp78 or P85 with BS‐I resulted in promotion of the migration and invasion of MHCC97L and HCCLM3 cells, compared with BS‐I treatment group. Importantly, the protein levels of phosphorylated AKT, phosphorylated GSK3β, phosphorylated S6, phosphorylated MEK1/2 and phosphorylated ERK1/2 were increased in MHCC97L and HCCLM3 cells, compared with BS‐I treatment group (Fig. 7C). Increased β‐catenin nuclear translocation (Fig. 7D) and decreased phosphorylated β‐catenin expression were found at the same time (Fig. 7C). Moreover, an increase in protein levels of uPA, MMP2 and MMP9 were found in Grp78 or P85 transfection and BS‐I treatment group, compared with BS‐I treatment group (Fig. 7C). These results indicated that overexpression of Grp78 or P85 rescues BS‐I‐mediated inhibition of migration and invasion of HCC cell.

## Discussion

Cancer metastasis is involved in cancer cell extravasation and subsequent invasion of surrounding tissue, which is regulated by cell surface mechanisms 36, 37. The identification of altered surface glycoproteins in the metastatic process contributes to reveal the mechanism of metastatic, discovery new biomarkers and for cancer diagnosis and explore new therapeutic targets. Recently, aberrant glycosylation of surface protein was reported. Yang *et al*. 38 reported that SNA‐I shows an enhanced binding to highly metastatic prostate cancer cells. Zhou *et al*. 39 found that lectin RCA‐I specifically bind to metastasis‐associated cell surface glycans in triple‐negative breast cancer. In this study, we found that lectin BS‐I bind to metastasis‐associated cell surface glycans in hepatocellular carcinoma cells.

Lectins were widely performed to investigate the alteration of protein glycosylation in cancers based on their ability to selectively recognize and reversibly bind to specific free sugars or glycans present on glycoproteins and glycolipids 40. On the other hand, lectins can affect vitality, metastatic and autophagy of cancer cells by modulating representative signalling pathways depending on their pharmacological activities 3. In this study, we found that treating MHCC97L and HCCLM3 cells with 1 or 4 μg/ml BS‐I significantly inhibits cell migration and invasion and down‐regulated the matrix metalloproteinase 2 (MMP2), matrix metalloproteinase 9 (MMP9) and urokinase‐type plasminogen activator (uPA) production. Further, AKT/GSK‐3β/β‐catenin pathway, the upstream of MMP2, MMP9 and uPA, was found to be involved in the inhibition of cell migration and invasion by BS‐I (Fig. 2). The expression of related molecules of RAS/RAF/MEK/ERK and integrin/FAK pathways was investigated by Western blot assay, and the results shown that the expression of integrinβ1, FAK and phosphorylated FAK was not changed after 1 μg/ml BS‐I and 4 μg/ml BS‐I treatment in MHCC97L and HCCLM3 cells. The result indicated that BS‐I inhibits migration and invasion of HCC cell is not *via* integrin/FAK pathways. In addition, BS‐I could not induce significant degradation of active Ras, phosphorylated B‐Raf and phosphorylated C‐Raf in MHCC97L and HCCLM3 cells. However, phosphorylated MEK1/2 and phosphorylated ERK1/2 were decreased with AKT/GSK‐3β/β‐catenin pathway inhibition. Thus, we concluded that BS‐I inhibits migration and invasion of HCC cell by suppressing AKT/GSK‐3β/β‐catenin pathway, because MEK1/2 and ERK1/2 are also regulated by of AKT 32. Further, we found that GSK3β inhibitor could rescue BS‐I‐mediated inhibition of cell migration and invasion and activate AKT/GSK‐3β/β‐catenin pathway (Fig. 3). In addition, these effects of BS‐I were mediated by inhibiting the activation of AKT/GSK‐3β/β‐catenin pathway and depended on specificity of lectin BS‐I binding to GalNAc (Fig. 3).

The glucose‐regulated protein (GRP78), also known as BiP/HSPA5, is first found to be a major regulator of endoplasmic reticulum (ER) stress signalling as an ER chaperone 10, 11, 12. Recently, increasing evidence supported that GRP78 could play critical roles in the resistance to chemotherapy agents, proliferation, invasion and metastasis of many human cancers 41, 42, 43, 44, 45. Moreover, a subfraction of GRP78 was found to preferential expressed at the surface of cancer cells 13, 14, 15, 46 and regulate signal transduction by forming complexes with specific cell surface proteins, such as α2‐macroglobulin (α2‐M*), Cripto and P85 19, 47, 48, 49, 50. Liu *et al*. 19 reported that surface GRP78 regulates PI3K/AKT signalling through direct complex formation with the p85. In this study, we identified GRP78 as a lectin BS‐I‐recognized membrane glycoprotein (Fig. 5) and found that lectin BS‐I interacts with GRP78, affects membrane localization of sGRP78 and attenuates the binding of sGRP78 and p85 to inhibit the activation of AKT/GSK‐3β/β‐catenin pathway (Fig. 6). Moreover, we found that overexpression of Grp78 or P85 could rescue BS‐I‐mediated inhibition of cell migration and invasion and activate AKT/GSK‐3β/β‐catenin pathway (Fig. 7).

In summary, our results demonstrated that lectin BS‐I binds to metastasis‐associated cell surface glycans and inhibits cell migration and invasion. This finding might further lead to the development of new therapeutic approaches targeted against HCC.

## Conflicts of interest

The authors declare that they have no potential conflict of interests.
